# Soft, Formstable (Co)Polyester Blend Elastomers

**DOI:** 10.3390/nano11061472

**Published:** 2021-06-01

**Authors:** Axel T. Neffe, Victor Izraylit, Paul J. Hommes-Schattmann, Andreas Lendlein

**Affiliations:** 1 Institute of Active Polymers, Helmholtz-Zentrum Hereon, 14513 Teltow, Germany; axel.neffe@hereon.de (A.T.N.); victor.izraylit@hereon.de (V.I.); p.hommes@gmx.net (P.J.H.-S.); 2 Institute of Chemistry, University of Potsdam, 14469 Potsdam, Germany

**Keywords:** thermoplastic elastomer, biomaterial, stereocomplexes, mechanical properties, form stability, crystallinity

## Abstract

High crystallization rate and thermomechanical stability make polylactide stereocomplexes effective nanosized physical netpoints. Here, we address the need for soft, form-stable degradable elastomers for medical applications by designing such blends from (co)polyesters, whose mechanical properties are ruled by their nanodimensional architecture and which are applied as single components in implants. By careful controlling of the copolymer composition and sequence structure of poly[(*L*-lactide)-*co*-(*ε*-caprolactone)], it is possible to prepare hyperelastic polymer blends formed through stereocomplexation by adding poly(*D*-lactide) (PDLA). Low glass transition temperature *T*_g_ ≤ 0 °C of the mixed amorphous phase contributes to the low Young’s modulus *E*. The formation of stereocomplexes is shown in DSC by melting transitions *T*_m_ > 190 °C and in WAXS by distinct scattering maxima at 2*θ* = 12° and 21°. Tensile testing demonstrated that the blends are soft (*E* = 12–80 MPa) and show an excellent hyperelastic recovery R_rec_ = 66–85% while having high elongation at break *ε*_b_ up to >1000%. These properties of the blends are attained only when the copolymer has 56–62 wt% lactide content, a weight average molar mass >140 kg·mol^−1^, and number average lactide sequence length ≥4.8, while the blend is formed with a content of 5–10 wt% of PDLA. The devised strategy to identify a suitable copolymer for stereocomplexation and blend formation is transferable to further polymer systems and will support the development of thermoplastic elastomers suitable for medical applications.

## 1. Introduction

Thermoplastic elastomers (TPE) combine processability from the melt and solution with (hyper)elastic recovery that is crucial, e.g., for applications, in which a polymeric material is exposed to repetitive mechanical stress [1]. Such situation can, e.g., occur in cardiovascular applications as in the substitutions of blood vessels or heart valves [2]. On the molecular level, polymer systems can show the desired properties when there are amorphous phases with low glass transition temperature (*T*_g_) that confer the flexibility of the system and physical netpoints that govern the form stability after elastic deformation. Such polymer structure is typically realized in block- and multiblock copolymers, in which different block types or segments show low miscibility [3,4]. Various polyurethanes [5], especially poly(ester urethanes) (PEU) [6,7] and poly(ether urethanes), are thermoplastic elastomers, in which usually the polyurethane part forms the physical netpoints. High deformability of such poly-meric materials requires a high content of the amorphous phase with low *T*_g_, while the form stability demands stable physical interactions. From a regulatory perspective, it would be advantageous for medical applications to use polymer components, which are already established clinical application. Two interesting components in this regard are poly(*ε*-caprolactone) (PCL) [8] and polylactide (PLA) [9]. PCL [10,11,12,13,14], PLA based on either [15,16] or both stereoisomers [17], as well as (poly[lactide-*co*-(ε-caprolactone)], PLC) copolymers [18] are components of clinically established devices.

The design of a suitable polymer system can now be based on the following criteria. (1) The generation of physical netpoints in a PLA containing polymer system can exploit the strong tendency of poly(*L*-lactide) (PLLA) to preferentially form crystallites with poly(*D*-lactide) (PDLA), the so called stereocomplexes (SC) [19,20,21], due to the lower rate of isotactic PLA crystallization [22]. The fast crystallization, high melting temperature, and high mechanical stability of SCs facilitate reduction of absolute crystallinity (*ϕ*_c_) and weight content of crystallites in comparison to homocrystallite-based systems without affecting the physical network stability [23]. Such stereocrystallites display, e.g., in PLLA/PDLA blends a lamellar triangular microstructure, with an edge length of ~350 nm [24], though confinement effects may lead to smaller crystallite sizes [23]. The formation of PLLA/PDLA stereocrystals can be analytically detected, e.g., by differential scanning calorimetry (DSC), as sufficiently large stereocrystallites show higher melting transitions ≥190 °C compared to the PLA homocrystallites (HC) (*T*_m_ ≤ 180 °C), and by wide angle X-ray scattering (WAXS), as distinctive signals at 2 θ = 12°, 21°, and 24° are observed that do not occur in the scattering of crystalline isotactic PLA [25]. (2) The stereocomplexes shall be formed by blending component A containing PLLA segments and polymer B containing PDLA segments. In this way, also the ratio of components can be used to tailor the polymer properties. Furthermore, such blend materials can be interpreted as self-reinforced composites [26], in which the nanosized filler is produced in situ. (3) The blend should show high deformability, which requires (a) a high weight percentage of an amorphous phase with a low *T*_g_ and (b) at least one component with a high weight average molar mass ≥140 kg·mol^−1^. The deformability of the polymer is in the first instance based on the decoiling of the polymer chains in the amorphous phase above their *T*_g_, which likewise entropy–elastic recoil after stress removal, which is enabled by the presence of the nanomorphology with physical netpoints. Plastic deformation of the materials is likely to be observed at high applied strains *ε*, while the increase of the overall crystallinity *ϕ*_c_ decreases the *ε* threshold value, at which irreversible restructuring of crystallites starts [27].

From these general design criteria, more detailed specifications can be deduced and compared to approaches in the literature. In terms of degradable thermoplastic elastomers based on stereocomplexes, the so far reported systems are PEUs containing PDLA or PLLA blocks [28,29], or ABA triblockcopolymers based on a soft polyester middle block and terminal PLLA or PDLA blocks [30,31]. While the systems work as designed, i.e., the *T*_m_ and crystallinity of the systems increased and led to an increase of the Young’s modulus *E*, the synthesis in all cases required multiple steps. Preferentially, the above-mentioned component A of our envisioned blend is a *L*-lactide containing copolyester that has a low *T*_g_, a high weight average molar mass *M*_w_, and has long enough PLLA segments to form stereocomplexes, while ideally showing only low tendency to form homocrystallites. A suitable comonomer for lactide, whose polymers have a *T*_g_ = 50 °C, is *ε*-caprolactone, as PCL has a low *T*_g_ of −60 °C, giving PLC copolymers. A PLC with a *T*_g_ ≤ 0 °C is expected to show an end temperature of the glass transition interval lower than room temperature and therefore such polymers are always in a rubber elastic state when handled and tested at room or body temperature. As PLC copolymers typically form a mixed amorphous phase [32], the theoretical glass transition temperature *T*_g,t_ can be calculated by the Fox-Equation [33] (Equation (1)). Here, *w*_1_ and *w*_2_ are the weight fractions of monomer 1 and monomer 2 and *T*_g1_ and *T*_g2_ are the glass transition temperatures of the corresponding homopolymers of monomer 1 and monomer 2, respectively:

(1) $$\frac{1}{T_{g,t}}=\frac{w_{1}}{T_{g_{1}}}+\frac{w_{2}}{T_{g_{2}}}$$

A *T*_g_ of the copolymer ≤ 0 °C is theoretically reached at a weight fraction of ≤65 wt% of LA (*T*_g,PLA_ = 50 °C) [34] and ≥35 wt% of CL (*T*_g,PCL_ = −60 °C) [35].

In fundamental studies it was shown that polylactide SC are formed when the monodisperse sequences contain at least seven repeating units, while for polylactide HC 11 units are required [36], though it is likely that in copolymers, due to the reduced chain mobility, longer sequences may be necessary [29]. The copolymerization of lactide and *ε*-caprolactone happens via ring-opening polymerization (ROP) of the corresponding lactones. When the employed catalyst is active in ROP as well as in transesterification, the reaction results in randomization of the sequence structure. The sequence structure will furthermore depend on the ratio of the comonomers and the polymerization conditions (temperature, time) and has to be closely monitored. If the average PLLA sequence length is too short, no stereocomplex crystals will form, while if it is too large, homocrystallization will be highly noticeable. In addition, caprolactone sequences may form crystallites, which is undesired in order to yield a large percentage of amorphous phase.

**Figure 1:**
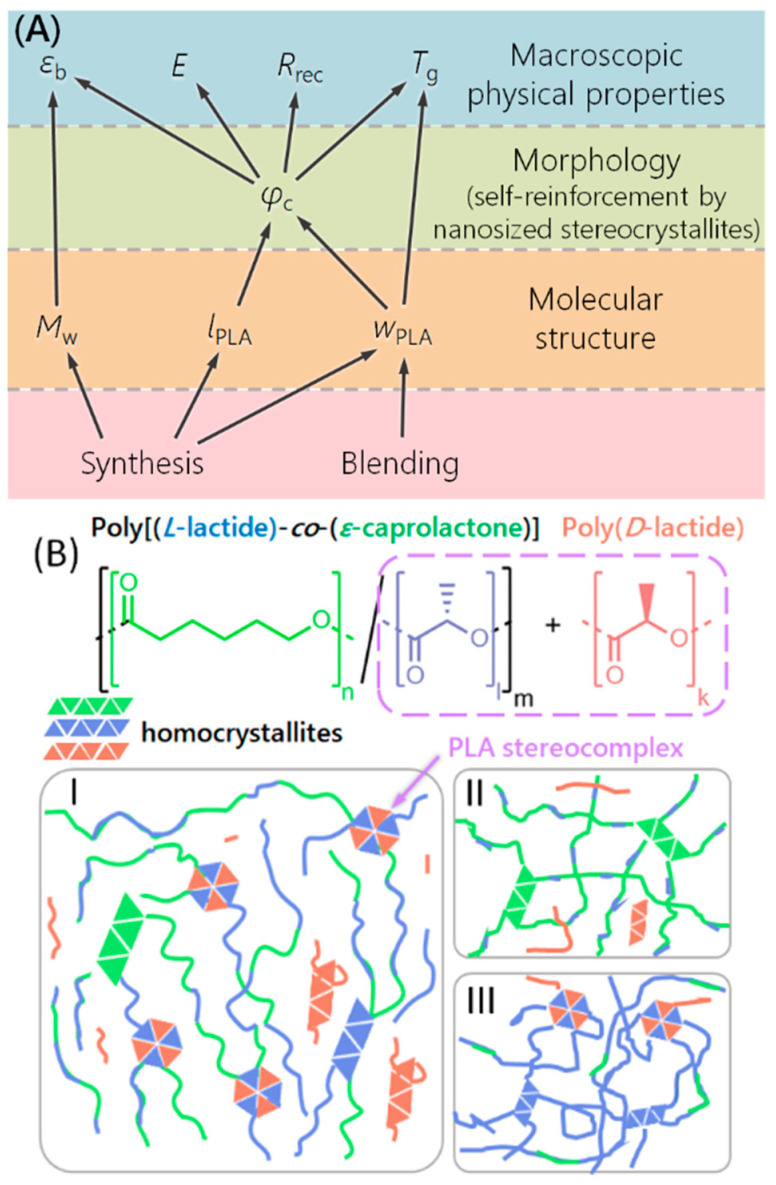
(**A**) Relationship between molecular structure (molar mass, length of lactide sequences, and lactide content) set by synthesis and blending, the material morphology (self-reinforcement by nanosized stereocrystallites), and the macroscopic material properties (elongation at break *ε*_b_, Young’s modulus *E*, form stability *R*_rec_, and glass transition temperature *T*_g_). Only a very precise molecular structure leads to a suitable material morphology (stereocrystal netpoints), which result in the display of targeted properties (low *T*_g_, combined with extendability and entropy–elastic recovery). (**B**) Chemical structures of the components of the envisioned blend and possible blend structures. (I) Balance between amorphous and (stereo)crystallizing regions. (II) System with too short lactide segment lengths unable to form homo- or stereocrystallites. (III) Too high lactide content is related to too high *T*_g_ and too high crystallinity for the envisioned mechanical behavior.

The overall relations between material characteristics ruled by synthesis and blending, the material morphology and the resulting material properties are depicted in Figure 1. The overall key aspect is the control of the morphology of the polymer blends on the nanolevel, which then rules the macroscopic properties of the material. 

In Figure 1B, the polymer structures as well as the schematic visualization of the polymer morphology at different lactide contents and sequence lengths are shown. Component B of the blend can be a PDLA homopolymer. It has been shown in the literature that the formation of stereocomplexes tends to be faster when one component has a disproportionally smaller molar mass than the other [37]. Therefore, the PDLA component was judged to be suitable when having an *M*_w_ ≤ 20 kg·mol^−1^.

In the following, first the influence of the synthesis conditions on the polymer composition, molar mass and sequence structure was investigated by GPC and NMR. By studying the thermomechanical properties (tensile tests and DSC) and wide-angle X-ray scattering of the PLC copolymers alone and in blends with PDLA, stereocomplex formation is shown for several blends, and structure–property relationships between polymer characteristics, polymer morphology and the polymer properties are established. The nontoxicity of the synthesized polymers is shown in an indirect cytotoxicity assay.

## 2. Materials and Methods

### 2.1. Chemicals

*L*,*L*-Dilactide (Purasorb L), *D*,*D*-dilactide (Purasorb D), and Poly(*D*-lactide, *M*_w_ = 147 kg·mol^−1^, Purasorb PD24) were bought from Corbion, Amsterdam, the Netherlands. *ε*-caprolactone (99%), 1-hexanol (99%, anhydrous), and 1-dodecanol were purchased from Acros Organics, Geel Belgium. Sn(Oct)_2_ (96%) was received from Alfa Aesar, Kandel, Germany. THF (anhydrous, >99.9%, 250 ppm BHT), DMSO-*d*_6_, and CDCl_3_ were from Sigma Aldrich, Munich, Germany, and chloroform (>99%) and methanol from Roth, Karlsruhe, Germany.

### 2.2. NMR

Spectra were recorded at room temperature on a DRX 500 Avance II spectrometer (500 MHz, Bruker, Rheinstetten, Germany; software Topspin version 1.3) using deuterated chloroform (CDCl_3_) or deuterated DMSO (DMSO-*d*_6_) as solvents.

### 2.3. FT-IR

Spectra were acquired on a Nicolet 6700 FTIR spectrometer (Thermo Fisher Scientific, Dreieich, Germany) in the range of 600–4000 cm^−1^, performing 100 scans per sample.

### 2.4. GPC

Molecular masses and their distribution of products were determined with a high-throughput gel permeation chromatography system, Tosoh EcoSEC HLC-8320 GPC including a refractive index detector (Tosoh Bioscience, Stuttgart, Germany) combined with a PSS Universal Data Center (PSS, Mainz, Germany), a viscometer ETA2010 (PSS), an EcoSEC UV detector 8320 (Tosoh Bioscience, Stuttgart, Germanyy), a light scattering detector SLD7100 (PSS) and two HT-GPC columns type PSS SDV analytical linear M 5 µm (PSS, Mainz, Germany) connected in series. Samples were measured in a concentration of 4 g·L^−1^ in chloroform (0.05 wt% toluene as internal standard, 35 °C, 1.0 mL·min^−1^) as eluent by universal calibration with polystyrene standards using WINGPC 6.2 (PSS) software.

### 2.5. DSC

DSC was performed using a Netzsch DSC 204 (Selb, Germany). Approximately 5 mg of the sample was analyzed in an aluminum pan. The experiments were conducted by heating the sample from −100 to 250 °C, then cooling to −100 °C, and again warming to 250 °C with a constant heating and cooling rate of 10 K·min^−1^. The glass transition temperature (*T*_g_) and the melting transitions were taken from the first heating run in order to evaluate the materials performance after film formation, i.e., handling after potential production.

### 2.6. Tensile Tests

Tensile tests were performed on a Z1.0 tensile test apparatus (Zwick GmbH, Ulm, Germany). The dogbone shaped samples with width of 2 mm and length of 20 mm were punched out of a solution-casted film (see: “Blending and film formation”) with thickness of 0.1–0.2 mm. The thickness of each sample was measured with a micrometer with 0.5 μm error and calculated as an average of three measurements at different positions. Stress–strain curves were acquired by stretching the samples in the dry state at ambient temperature and a constant deformation rate of 10 mm·min^−1^ until breakage occurred. Elongation *ε*_b_ and stress *σ*_max_ were recorded. Young’s modulus *E* was calculated as the slope of the initial linear segment of σ(ε) curves, typically at *ε* = 0–0.5%. The values were calculated as average of three measurements. In one set of examples, samples were stretched to defined strains and the recovery was recorded to determine the recovery rate *R*_rec_ according to Equation (2):

(2) $$R_{\mathrm{rec}}=\frac{\varepsilon -\varepsilon_{\mathrm{rec}}}{\varepsilon }\cdot 100\%$$

with *ε* = elongation (strain) = $\frac{l_{\varepsilon }-l_{0}}{l_{0}}$·100%, with *l_ε_* = length after elongation, and *l*_0_ = original length, and *ε*_rec_ = elongation after the recovery.

### 2.7. WAXS

WAXS measurements were conducted with a D8 Discover spectrometer with a 2D-detector from Bruker AXS (Karlsruhe, Germany) with Cu K_α_ radiation (λ = 0.154 nm) at a voltage of 40 kV and a current of 40 mA at room temperature. Peak position was determined with Δ*θ* = 0.1° error originating from variations in sample thickness and position in the sample holder.

### 2.8. Synthesis of Poly([(L-lactide)-co-(ε-caprolactone)] (PLC)

The exact details of the different synthesis approaches are summarized in Table 1. In a typical example, ε-caprolactone (CL) was purified by vacuum distillation using a Vigreux column (85 °C, 5 mbar). LA (60.0 g, 416.3 mmol) and CL (38 mL, approximately 40 g, 350 mmol) were mixed in a dry 500 mL three-neck-flask under argon. The flask was heated to 140 °C while stirring the mixture with a magnetic stirrer. After 5–10 min a colorless melt was obtained. Then, Sn(Oct)_2_ (125 mg, 0.31 mmol, in about 5.1 mL of anhydrous THF) was added dropwise through a rubber septum. The resulting mixture was stirred for 53 h at 140 °C. After cooling to room temperature, 400 mL of chloroform were added and the mixture was stirred for at least 8 h. The supernatant was collected, additional 400 mL of chloroform were added to the residue and the stirring/dissolution steps were repeated until the crude polymer was completely dissolved into 2000 mL of chloroform. The unified solutions were vigorously mixed for 15 min and then precipitated in methanol in 250 mL portions, with 2.5 L of methanol as precipitating agent for each portion. The precipitate was washed with about 2 L of methanol and was dried in a vacuum oven at 60 °C for at least 1 day. The yield was up to 90 g of copolymer.

Characterization (blend from several syntheses): ^1^H NMR (500 MHz, CDCl_3_): δ = 5.23–5.16 (q, CH, LA-LA diad, 2.83 H), 5.20–5.09 (q, CH, LA-CL diad; partially overlapping with LA-LA diad, ~1 H), 4.21–4.11 (m, O-CH_2_, CL-LA diad, 0.99 H), 4.11–4.03 (m, O-CH_2_, CL-CL diad, 2.04 H), 2.48–2.36 (m, CO-CH_2_, LA-CL diad, 1H), 2.36–2.28 (m, CO-CH_2_, CL-CL diad, 2.06 H), 1.76–1.34 (m, CH_2_ and CH_3_, 21.14 H) ppm. Ratio LA-LA to LA-CL diads: 3.4:1 (target: >2.7), molar ratio LA:CL: 71:29 (target: 72:28 ± 1). ^13^C NMR (125 MHz, CDCl_3_): δ = 173.6, 172.9, 170.3, 169.7, 69.1, 68.3, 65.4, 64.1, 34.2, 32.8, 28.5, 25.7, 24.7, 16.7 ppm. GPC (CHCl_3_, universal calibration): *M*_n_ = 61 kg·mol^−1^, *M*_w_ = 159 kg·mol^−1^, *D* = 2.5, *T*_g_: −1 °C. FT-IR: 2990, 2932, 2865, 1753, 1731, 1455, 1388, 1352, 1179, 1125, 1090, 1045 cm^−1^.

### 2.9. Synthesis of Poly(D-lactide) with an M_n_ of 16 kg·mol^−1^ (PDLA 16k)

PDLA 16k was synthesized by mixing *D*,*D*-dilactide (100.0 g, 694 mmol) and 1-hexanol (682 mg, 6.67 mmol) in a dry 500 mL three-neck-flask under argon. While stirring at 135 °C, Sn(Oct)_2_ (112 mg, 0.278 mmol in 4.6 mL of anhydrous THF) was added dropwise. The resulting mixture was stirred for 1 h at 135 °C. After cooling to room temperature, 400 mL of chloroform was added and the mixture was stirred until complete dissolution of the crude product. The obtained solution was precipitated in portions of about 200 mL by addition to cold methanol (2 L each). The precipitate was collected and washed with about 1 L of methanol. The precipitate was dried in a vacuum oven (membrane pump) at 60 °C. The yield was up to 95 g of polymer.

Characterization: ^1^H NMR (500 MHz, CDCl_3_): δ = 5.14–5.02 (q, CH, 1H), 1.58–1.42 (d, CH_3_, 3H) ppm (signals of 1-hexanol near the noise). ^13^C NMR (125 MHz, CDCl_3_): δ = 169.7, 69.1, 16.7 ppm. GPC (CHCl_3_, universal calibration): *M*_n_ = 16.2 kg·mol^−1^, *M*_w_ = 18.0 kg·mol^−1^, *D* = 1.11. *T*_g_: 49 °C, *T*_m_ = 173 °C. FT-IR: 3003, 2939, 1749, 1458, 1378, 1363, 1211, 1182, 1131, 1084, 1043 cm^−1^.

### 2.10. Blending and Film Formation

In total, 600–700 mg of PLC and PDLA-16k in the desired ratio were dissolved in 12 mL of CHCl_3_ (58 °C, thermomixer; 700 rpm, 2 h). The solution was cooled to room temperature and poured into a PTFE evaporation dish. The dish was covered tightly with aluminum foil and left for evaporation of the solvent in the hood for at least 1 day. The film was further dried at 60 °C in a vacuum oven (membrane pump, overnight). The film was cooled to room temperature and the removed from the dish using tweezers.

## 3. Results and Discussion

While the design criteria of the polymer system detailed in the introduction gave clear specifications regarding the targeted copolymer composition, such as that the amorphous phase of the PLC copolymer should not contain more than 65 wt% lactide to reach the desired *T*_g_ ≤ 0 °C, a series of syntheses were required to identify suitable feeding ratios of the comonomers and polymerization conditions. First, it has to be considered that the composition of the feed during the synthesis is not necessarily the same as the composition of the synthesized copolymer, as some starting material may not be incorporated into the polymer. In addition, crystallized segments in the polymer chain do not contribute to the *T*_g_ as the composition of the amorphous phase changes. The employed Sn(Oct)_2_ catalyzes transesterification at elevated temperatures and longer reaction times [38] alongside with ROP, which was desired in this work. Due to the lower reactivity of CL in comparison to LA [39], copolymerization without transesterification, as can be received with other catalysts [40] or by adding a transesterification inhibitor [41], would give gradient or block copolymers. CL end groups are promoting transesterification more than LA end groups [42] so that transesterification increases compared to ROP at high conversions and with prolonged reaction time. The molar mass, molar mass distribution, the comonomer fractions and the sequence structure of the copolymer were determined by gel permeation chromatography (GPC) and nuclear magnetic resonance spectroscopy (NMR). The performed syntheses are summarized in Table 1.

The lower reactivity of the CL monomer compared to LA means that at 140 °C reaction temperature at least 24 h of reaction time were required to achieve nearly complete conversion of both monomers. The molar composition of the copolymer is reasonably similar to the feed ratio only at such high conversions. In the course of the project, a series of PLC with a 70:30 and a 67:33 feed were synthesized several times. Analyses showed a high reproducibility of the produced polymers in terms of composition, sequence structure, and molar mass. The molar ratio of LA and CL in the copolymer can be determined from the ratio of the integrals in the ^1^H NMR spectra representing the C*H* of the LA units compared with the –OC*H*_2_- or –CO-C*H*_2_- signals (see Supplementary Materials Figure S1). Furthermore, the ratio of LA-LA dyads to LA-CL dyads is available through comparing the integrals of the indicated signals. While the signals of LA-LA and LA-CL dyads partially overlap, the LA-CL and CL-LA dyads have to have the same integral for the investigated system. As the CL-LA and CL-CL dyads are better separated than the LA-LA and LA-CL dyads, the LA-CL value can be taken for the calculation. The information on the relative dyad content together with the molar ratio of lactide and caprolactone can be used to determine the number average sequence length of lactide and caprolactone blocks as well as the randomness of the copolymer [43]. For this purpose, the Equations (3)–(6) [44] are employed

(3) $$l_{\mathrm{LA}}=\frac{2\left(\mathrm{LA}\right)}{\left(\mathrm{LA}-\mathrm{CL}\right)}$$

(4) $$l_{\mathrm{CL}}=\frac{2\left(\mathrm{CL}\right)}{\left(\mathrm{LA}-\mathrm{CL}\right)}$$

(5a) $${\left(l_{\mathrm{LA}}\right)}_{\mathrm{random}}=\frac{1}{\left(\mathrm{CL}\right)}$$

(5b) $${\left(l_{\mathrm{CL}}\right)}_{\mathrm{random}}=\frac{1}{\left(\mathrm{LA}\right)}$$

(6) $$R=\frac{\left(\mathrm{LA}-\mathrm{CL}\right)}{2\left(\mathrm{LA}\right)\left(\mathrm{CL}\right)}$$

with (LA) and (CL) being the monomer molar fractions in the copolymer, and (LA − CL) the relative molar fraction of this dyad. *l*_LA_ and *l*_CL_ are the number average block length of these comonomers, (*l*_LA_)_random_ and (*l*_CL_)_random_ are the Bernoulli average block lengths, and *R* is the randomness character of the polymer, which is defined as the ratio of the Bernoulli block length and the observed block length. A totally random copolymer has the randomness of 1, a block copolymer approaches 0, while copolymers with not completely random distribution are between 0 and 1. Here, all syntheses were stopped before a completely random distribution of repeating units was reached. The randomness character increased with prolonged reaction times, highlighting the occurrence of transesterification. Higher temperatures generally increased the rate of reaction, but also that of transesterification. High *M*_w_ ≥ 140 kg·mol^−1^ values, potentially contributing to material elasticity, were reached only in the absence of a co-initiator. Even the purified educts and catalyst contain enough residual nucleophiles such as water to initiate the ROP.

Table 2 summarizes the thermal transitions, crystallinities, and mechanical properties of selected synthesized copolymers. PLC-84-24.0-49, PLC-82-21.1-51, PLC-57-5.9-56, and PLC-57-4.9-103 were judged to be unsuitable for the further studies due too low *M*_w_ and/or absence of PLA crystallization and were therefore not investigated in tensile tests (see data in Supplementary Materials Table S1). The desired *T*_g_ ≤ 0 °C of the copolymer was received when the lactide content of the feed was ≤70 mol%. The determined glass transition temperatures were in the range as calculated by the Fox equation assuming a totally amorphous polymer, though, as planned, generally a bit lower due to polylactide crystallization, which decreases the lactide content in the amorphous phase. The polymers with an *l*_LA_ ≥ 4.8 formed polylactide crystallites. The crystallinity of the samples increased with increase of *l*_LA._ The melting temperature region was broad (~70–150 °C), corresponding to small crystallites of varied size, or phase dilution. As discussed above, in monodisperse lactide homopolymers a sequence length of at least 11 units is required to allow for homocrystallization [36]. The here determined number average sequence length allowing crystallization is lower than 11, as *l*_LA_ is averaged and also represents sequences longer than the average of 4.8. Caprolactone segment crystallization was observed in some samples with a number average sequence length *l*_CL_ > 2.2, again with a broad melting transition region (*T*_m_ ~ 40–55 °C) of low melting enthalpy. In the literature, a calculated *l*_CL_ of ≥4.91 was required to observe spontaneous PCL segment crystallization in a random copolymer with much higher CL contents, though isothermal crystallization could also be induced at lower sequence lengths [45]. High crystallinity and low molar mass generally correlated with lower elongation at break (ε_b_) and higher Young’s modulus *E* for the copolymers (see also discussion of general trends at the end of the results and discussion section).

In the next step, the ability of PLCs with different molar compositions and sequence structure to form stereocomplexes in blends with PDLA of *M*_w_ = 16 kg·mol^−1^ according to the design as stated in the introduction was evaluated. To highlight the easier formation of SC with PDLA of lower mass, in one example a PDLA with an *M*_w_ of 147 kg·mol^−1^ was used.

Table 3 summarizes the overall lactide content, thermal transitions, crystallinity, and mechanical properties of the blends. The relative degree of crystallinity was estimated in the following way: for the estimated maximum possible stereocomplex formation, a 1:1 (L-LA):(D-LA) composition of the stereocomplexes was assumed. It is known that stereocomplexes also form outside this ratio [46], but for a qualitative understanding of our system, this approximation can be taken as valid. Any LA that was not engaged in SC formation was assumed to be available for homocrystallization, enabling calculation of *χ*_c,LA_ and the overall crystallinity *ϕ*_c,LA_ according to Equations (7)–(9).

(7) $$\chi_{c,\mathrm{LA}}SC=100\cdot \;\frac{\Delta H_{m,\mathrm{SC}}}{\Delta H_{m,\mathrm{SC}}^{0}\cdot 2w_{\mathrm{PDLA}}}$$

(8) $$\chi_{c,\mathrm{LA}}HC=100\cdot \;\frac{\Delta H_{m,\mathrm{HC}}}{\Delta H_{m,\mathrm{HC}}^{0}\cdot \left(w_{\mathrm{PDLA}}+\left(1-w_{\mathrm{PDLA}}\right)\cdot w_{\mathrm{LA}\left(\mathrm{PLC}\right)}-2w_{\mathrm{PDLA}}\cdot \frac{\chi_{c,\mathrm{LA}}\mathrm{SC}}{100}\right)}$$

(9) $$\phi_{c,LA}=100\cdot \frac{\Delta H_{m,\mathrm{SC}}}{\Delta H_{m,\mathrm{SC}}^{0}}+100\cdot \frac{\Delta H_{m,\mathrm{HC}}}{\Delta H_{m,\mathrm{HC}}^{0}}$$

with *χ*_c,LA_: relative crystallinity, *ϕ*_c,LA_: absolute crystallinity, Δ*H*_m,SC_: melting enthalpy of the stereocomplexes, Δ*H*_m,HC_: melting enthalpy of the homocrystallites, $\Delta H_{m,\mathrm{SC}}^{0}$: melting enthalpy of 100% crystalline PLA SC; $\Delta H_{m,\mathrm{HC}}^{0}$: melting enthalpy of 100% crystalline PLA HC, *w*_PDLA_: weight fraction of PDLA in the blend, *w*_LA(PLC)_: weight fraction of LA in the PLC copolymer.

**Figure 2:**
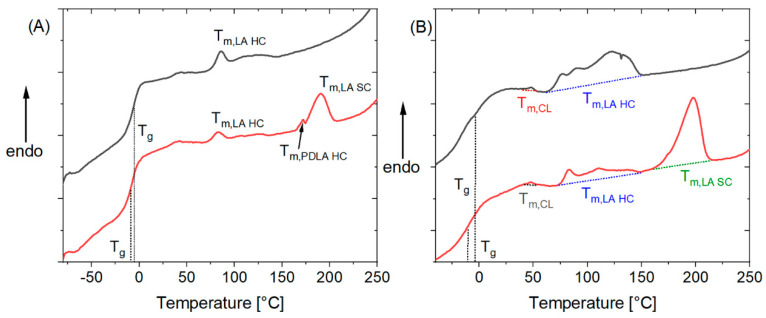
(**A**) Comparison of DSC thermograms (1st heating run) of PLC-58-4.84-156 (black) and the blend B95-PLC-58-4.8-156 (red). In the blend, a second melting transition indicative for stereocomplex formation was observed. Higher lactide crystallinity of the blend sample leads to reduction of *T*_g_. (**B**) Comparison of DSC thermogram (1st heating run) of PLC-62-7.2-180 (black) and the blend B95-PLC-62-7.2-180 (red). Visible in this representative DSC is the typical broad melting transition of lactide homocrystallites observed in the copolymers and the very small CL melting peak.

Figure 2 and Supplementary Materials Figure S2 show typical examples of DSC thermograms of the synthesized and blended PLC. Stereocomplex formation was assumed when a melting transition of ~80–175 °C originally observed in PLC was shifted to higher *T*_m_ ranges > 190 °C as the result of blending with PDLA. It should be noted that melting temperatures of polymer crystallites are size dependent, and decrease with decreasing lamellar thickness [47,48]. Therefore, small PLA stereocomplex crystallites may show lower melting transitions than the standard > 190 °C [49]. Therefore, in addition to the DSC experiments, WAXS studies were performed, which allowed unambiguous identification of whether PLA stereocomplexation took place (see below). All samples with an *l*_LA_ of ≥4.8 formed stereocomplex crystallites. This is the same number average sequence length as observed for the formation of homocrystallites, despite literature reports that a longer sequence length of oligolactide is required for homo- than for stereocrystallization [36]. As discussed above, the *l*_LA_ is only indicative for sequence length, as there is a distribution of sequence lengths in the synthesized polymers. There is a strong, but not linear correlation between sequence length and crystallinity of the samples (Supplementary Materials Figure S3), which is observed for the relative crystallinity in the copolymers (*χ*_C,LA_ HC), the relative crystallinity of stereocrystallites in the blends (*χ*_C,LA_ SC), and the absolute crystallinity of the blends (*ϕ*_C,LA_). The occurrence of an SC-related additional melting transition at >190 °C led to a reduction of the melting enthalpy of the peak attributed to the homocrystallites, as can be exemplarily seen in Figure 2B. This indicates that oligolactide segments that form homocrystallites in the absence of PDLA are now engaging in the formation of stereocomplexes. In some cases, the amount of homocrystallites found was very small (B80-PLC-65-10.8-80, B90-PLC-56-5.4-145, B95-PLC-58-4.8-156). This correlates with a high PDLA content or a short *l*_LA_. Both parameters seem to support SC formation over HC formation. The melting temperature, which is a qualitative measure of crystallite size [47,48,50], increased with increasing *l*_LA_, which indicates that larger oligolactide sequences lead to the formation of larger crystallites. The overall melting enthalpy showed a weak linear correlation with the LA content (R^2^ = 0.70) in the blends, while the crystallinity showed even weaker linear correlation with the LA content (R^2^ = 0.57 for the copolymers and R^2^ = 0.38 for the blends), suggesting that further structural aspects, likely *l*_LA_, were playing a role in the crystallization process (see Supplementary Figure S4). In some samples, in addition to the broad HC melting transition, a sharper melting peak at ~170 °C was observed (compare Figure 2A). It is likely that this peak can be attributed to PDLA HCs. Comparing first and second heating runs for PLC, the *T*_g_ of the second heating run was higher than of the 1st heating run, as the crystallized LA did not contribute to *T*_g_ associated in the mixed phase in the first run. The cooling regime did not allow recrystallization of the molten LA so that in the second heating either only an amorphous phase was present, or crystallization occurred during heating with a melting transition temperature consistent with homocrystallites (i.e., *T*_m_ < 180 °C). In addition, the *T*_g_ of the blends was in most cases lower than that of the PLC, despite the higher overall lactide content. The overall higher LA crystallization in these cases reduces the LA content in the mixed amorphous phase leading to this effect. Such lowering of *T*_g_ through SC formation indirectly contributes to material elasticity. The sample formed with PDLA 147k had lower relative SC crystallinity than the corresponding sample with PDLA 16k, indicating the easier formation of SC when one component is of lower molar mass.

**Figure 3:**
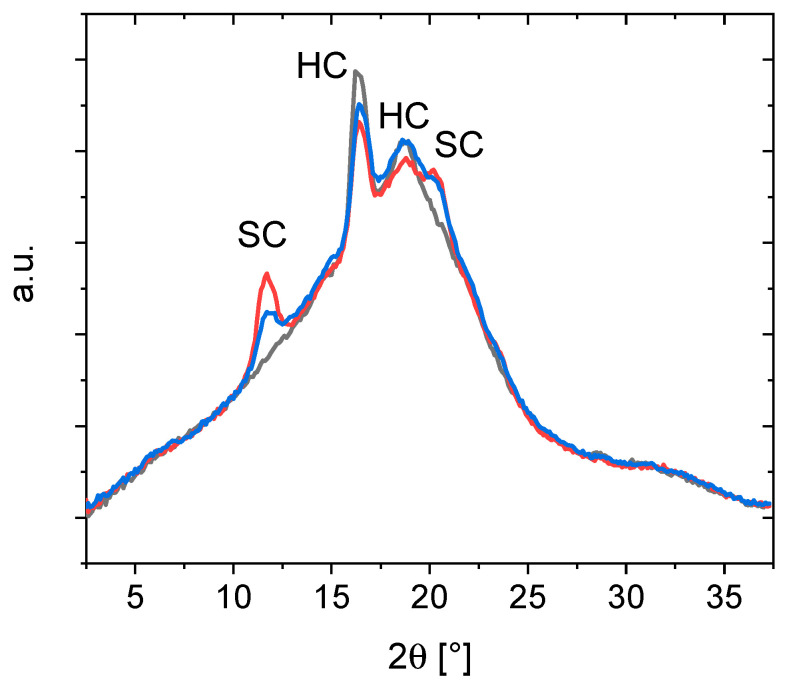
WAXS spectra of PLC-58-4.8-156 (black) and the blends B95-PLC-58-4.8-156 (blue) and B90-PLC-58-4.8-156 (red). Only in the blend, peaks indicative for stereocomplex (SC) formation are observed. Homocrystallite peaks occur in the copolymers as well as in the blends. The SC peaks increase with the PDLA content.

In Figure 3 and Supplementary Materials Figure S5, typical WAXS spectra of a PLC and its PDLA16k blends are depicted. In all spectra, peaks at 17° and 19° indicative for PLA homocrystallites (HC) are present [25]. In some samples, additional peaks at 15° and 22.5°, likewise attributed to homocrystallites, occurred. Only in spectra of blends, furthermore peaks at 12°, 21°, and in some samples 24°, were observed, which show the presence of PLA stereocrystallites (SC) [25]. The peak intensity increased with the amount of PDLA in the blend. In all cases, these peaks occurred only for samples that also showed a *T*_m_ > 190 °C. It can thus be concluded that in the investigated systems the spontaneous formation of SC in the blends leads to formation of crystallite sizes that have a melting transition typically associated with SC. Though there was some stereocomplex formation observed with PDLA 147k, the melting enthalpy was low and the WAXS peaks had low intensity. When testing commercially available PLC with a 79:21 mol% LA:CL, no stereocomplex formation with PDLA 147k was observed despite a *l*_LA_ of 7.0 (data not shown). This may suggest that the minimum sequence length required for the formation of stereocomplexes may also depend on the chain mobility during the mixing, and therefore a higher required *l*_LA_ when the PDLA *M*_n_ increases. An alternative explanation may be the changed miscibility of the two compounds when increasing the molar mass of PDLA. It has to be pointed out that at such high LA content of the copolymer, the material is below its *T*_g_ at room temperature and therefore not soft and (hyper)elastic as desired. Stereocomplex formation in non-soft copolymer blends has been observed for PLC based on *D*-lactide blended with PLLA [51]. In case of PDLA 16k, generally the melting enthalpies were higher and signals in the WAXS more prominent. The higher the content of PDLA in the mixture, the higher was also the content of stereocomplexes.

**Figure 4:**
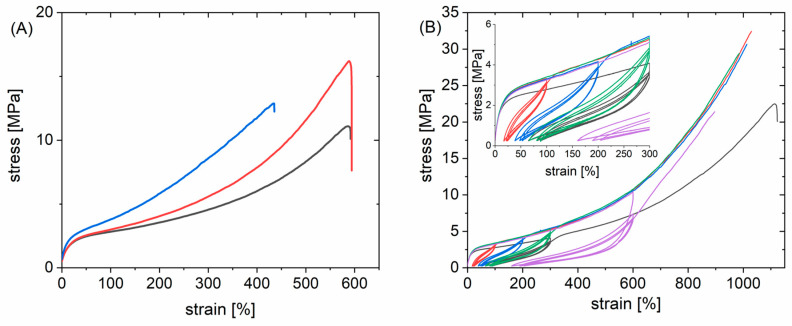
(**A**) Tensile curves of PLC-56-5.5-182 (black) and its blends B95-PLC-56-5.5-182 (red) and B90-PLC-56-5.5-182 (blue). (**B**) Tensile curve of PLC-56-5.4-145 (black) and its B95-PLC-56-5.4-145 blend after prior extension to 100 (red), 200 (blue), 300 (green), or 600% (purple). The initial extension did not lead to changes in tensile behavior on further extension.

**Figure 5:**
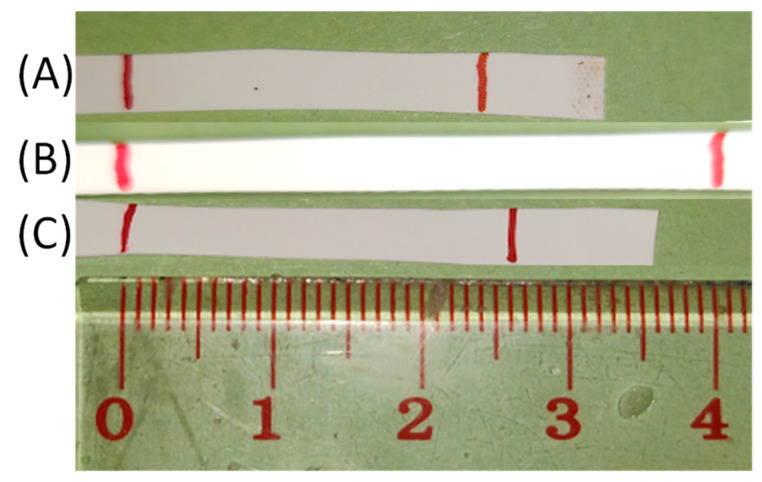
Stretching and recovery of the B90-PLC-62-7.2-180 blend. (**A**) After 1st stretching by 100%, (**B**) stretched, (**C**) recovered after 10th stretching.

The mechanical properties of the PLCs and blends were determined in tensile tests at room temperature. Representative curves are shown in Figure 4 and Supplementary Materials Figure S6. The profile of the tensile curve was similar for the copolymers and their blends. In general, the blends had higher Young’s moduli *E* and lower elongation at break *ε*_b_ than the corresponding PLC alone. Such behavior was also observed for other stereo-complex-based TPEs [30,31]. In the case of a 95:5 mixture, the reduction of *ε*_b_ was only slight, while for some 9:1 and all 4:1 mixtures the reduction compared to the PLC component alone was notable. The *ε*_b_ tended to be higher for all blends based on a PLC with a *M*_w_ > 140 kg·mol^−1^ compared to the systems based on PLC with lower *M*_w_. *E* generally increased with the PDLA content of the blend. All of this suggests to use a low PDLA weight content in the blend in order to maximize *ε*_b_ and reduce *E*. Good correlations were observed for the absolute crystallinity with the *ε*_b_ and the Young’s modulus (see Supplementary Materials Figure S7). *ε*_b_ had an inverse linear correlation with the absolute crystallinity of the copolymers (R^2^ = 0.76) as well as of the blends (R^2^ = 0.51), i.e., *ε*_b_ increased with decreasing ϕ_c,LA_. Generally, this is to be expected [52]. In order to receive a material with high extendability, nonlinear stress–strain behavior, and high shape recovery *R*_rec_, i.e., a hyperelastic material, it was therefore of interest to reduce the crystallinity, which is why the developed relationships of sequence length and crystallite formation are so relevant. Young’s modulus and absolute crystallinity had an exponential relation (R^2^ = 0.998 for PLC, and 0.86 for the blends), as was also observed for other semicrystalline systems [53]. Stretching and relaxation to different strains showed a small hysteresis of extension and recovery curves as well as a small permanent deformation in the first extension and very low further permanent deformation in subsequent experiments (see also Figure 5) [54]. This means that the blends behaved as semicrystalline polymers that show plastic deformation of crystals at the investigated strains [23] and highest *ε*_b_ and form stability when the crystallinity is very low. *R*_rec_ values determined from the experiment shown in Figure 4b and from manual experiments documented by photos (such as Figure 5 and Supplementary Materials Figure S8) were in the range of 66–85% for the blends already in the first cycle. This is a bit higher than typically observed in physical networks such as multiblock copolymers (R_rec_ = 55–85%) [55], thermoplastic polyurethanes (R_rec_ ~ 70%) [56]; (R_rec_ = 47–80%) [57], poly(butylene 1,4-cyclohexanedicarboxylate) (R_rec_ = 11–64%) [58], or poly(butylene terephthalate)/ethylene rubber blends (R_rec_ ~ 60%) [59]. In PCL-PLLA multiblock copolymers blended with PDLA, only at crystallinities below 6% and limited strains full form stability was found [23]. In most samples investigated here, this crystallinity was exceeded by far, except for B95-PLC-58-4.8-156, while B90-PLC-56-5.4-145 and B90-PLC-62-7.2-180 were close to this mark, and all of these samples showed low *E* and high *ε*_b_. In this way, B95-PLC-58-4.8-156 represents the material composition fitting to our design best. Altogether, the blends demonstrated excellent hyperelasticity at low *E*, demonstrating the design to be successful and showing that only by the conducted systematic investigations of the polymer system relationships between the different hierarchical levels of the material system as shown in Figure 1A can be established.

Figure 5 shows the extensibility and entropy–elastic recovery of the B90-PLC-62-7.2-180 blend, demonstrating an easy extendability and form recovery after stress removal, which is highly remarkable for PLC copolymers and for polymers with high lactide content. In comparison, a blend of a PLC copolymer with too high lactide content, which is related to a higher *T*_g_ (28 °C) and high crystallinity shows a much higher Young’s modulus (910 ± 167 MPa) and poor shape recovery (Supplementary Materials Figure S8).

The material was hierarchically designed in order to set the macroscopic properties of the polymer by controlling the morphology on the nanolevel. As specified in the introduction, the final macroscopic properties with high extendability and form stability are of relevance in biomedical applications. In order to show that the material principally is suitable for such an envisioned application, we tested both components of the blend, PLC and PDLA16k, for cytotoxicity in an indirect contact test using L929 mouse fibroblasts (see Supplementary Materials, including Figures S9–S14). The overall cell viability after 48 h (97.3%) was similar to the control (97.7%), while the absolute number of cells was slightly reduced (by 31%). Cell morphology was unchanged compared to the control. The cells formed a tight layer, and no cell lysis was observed. LDH release was unchanged, demonstrating intact cell membranes, while the mitochondrial activity was reduced slightly by 16%. Furthermore, the endotoxin content of the samples was <0.06 EU·mL^−1^, so that the materials also passed the strict limits for biomaterials in contact with cerebrospinal fluids. Therefore, the materials passed this fundamental toxicity evaluation, opening the possibility for further exploration in specific biomedical applications.

## 4. Conclusions

Altogether, we could demonstrate a sequence depending ability of PLC copolymers to form lactide HC and SC, as envisioned in the design strategy. Therefore, the mechanical properties of the blends could be controlled by the formation of nanosized netpoints, formally constituting a self-reinforced composite. It is possible to synthesize PLC with the desired ability to form SC and a low *T*_g_ so that the copolymers and its blends are hyper-elastic at room temperature. The high elongation at break could in this way be combined with excellent form stability of the material, which is highly unusual for polymers with high lactide content. The key to realize the desired macroscopic performance of the blends was the control of the morphology of the polymer blends on the nanolevel. The relative percentage of stereocrystallites compared to homocrystallites can be increased in systems with high PDLA content and short *l*_LA_. Such a structure, with an overall low crystallinity, allowed for the highest elongation at break and lowest Young’s modulus in the investigated samples and represents the designed and desired morphology best. The general design strategy for partially randomized copolymers is likely transferable to other copolymer systems and therefore of general value in the design of TPE. PLC/PDLA mixtures can be processed from solution, as in solution no stereocomplexes form. The SC are formed spontaneously at room temperature by removing the solvent. Potentially, the materials as TPE are reshapable. In contrast to the known (multi)block copolymers stabilized by stereocomplexes, no multistep synthesis is required, drastically facilitating the production and broaden the applicability of a copolymer that is established in clinical applications. The fundamental studies showing the nontoxicity of the system further support the drive of applying the presented polymer system in such applications.

## 5. Patents

ATN, PJHS, AL are co-inventors on patent application related to the presented polymer system.

## Figures and Tables

**Table 1:** Influence of feed composition, temperature, and reaction time on the composition, sequence structure, and *T*_g_ of the copolymer.

Sample ID	Feed	Reaction Time	Conversion		Comp.	Sequence Structure			Molar Masses and Distribution		
	LA:CL		LA	CL	LA:CL	*l*_LA_	*l*_CL_	*R*	*M*_n_ (kg·mol^−1^)	*M*_w_ (kg·mol^−1^)	*Đ*
	(mol%)	(h)	(%)	(%)	(mol%)						
PLC-84-24.0-49 ^a^	75:25	48	99	93	89:11	24.0	3.0	0.38	26	49	1.88
PLC-82-21.1-51 ^b,d^	75:25	44	99	96	88:12	21.1	2.9	0.39	35	51	1.46
PLC-57-5.9-56 ^a^	70:30	48	99	99	68:32	5.9	2.8	0.53	30	56	1.86
PLC-65-10.8-80 ^b,e^	70:30	44	99	96	75:25	10.8	3.6	0.37	62	80	1.29
PLC-68-10.6-71 ^b,f^	70:30	5	98	90	77:23	10.6	3.2	0.41	37	71	1.92
PLC-57-4.9-103 ^b,f^	70:30	21	98	99	68:32	4.9	2.3	0.64	59	103	1.75
PLC-56-5.4-145 ^c,e^	70:30	53	98	98	67:33	5.4	2.6	0.57	70	145	2.1
PLC-58-4.8-156 ^c,e^	69:31	26	95	98	69:31	4.8	2.2	0.67	67	156	2.3
PLC-62-7.2-180 ^c^	67:33	23	nd	nd	72:28	7.2	2.8	0.50	76	180	2.4
PLC-56-5.5-182 ^c^	67:33	48	nd	nd	67:33	5.5	2.7	0.55	80	182	2.3
PLC-53-4.4-103 ^b,e^	66:34	24	98	99	64:36	4.4	2.5	0.64	66	103	1.56
PLC-50-3.9-101 ^b,e^	66:34	27	98	99	61:39	3.9	2.5	0.66	64	101	1.58
a54-PLC-62-6.6-159 ^e^	70:30	53	nd	nd	72 ± 1	6.6	2.6	0.54	61 ± 17	159 ± 68	2.5 ± 0.5
a9-PLC-56-6.4-149 ^e^	67:33	53	nd	nd	69 ± 2	6.4	2.9	0.51	64 ± 17	149 ± 64	2.3 ± 0.6

Nomenclature PLC-X-Y-Z: X: wt% LA content, Y: *l*_LA_ (number average oligolactide sequence length), Z: *M*_w_. aW-PLC-X-Y-Z: average values from W syntheses. a: 1-Hexanol as co-initiator, 120 °C, b: 1-Dodecanol as co-initiator, c: no co-initiator added. d: 120 °C, e: 140 °C, f: 170 °C, nd: not determined. Comp: composition as molar ratio, *l*_LA_: number average oligolactide sequence length (based on single repeating units), *l*_CL_: number average oligocaprolactone sequence length, *R*: randomness, defined as Bernoullian number average seq. length/observed sequence length, *M*_n_: number average molar mass, *M*_w_: weight average molar mass, *Đ*: dispersity.

**Table 2:** Composition, thermal transitions, crystallinity, and tensile properties of selected synthesized PLC.

Sample ID	DSC					Tensile Properties		
	*T*_g_ (°C)	*T*_m_ (°C)	Δ*H*_m_ (J·g^−1^)	*χ*_c,LA_ (%)	*ϕ*_c,LA_ (%)	*E* (MPa)	*σ*_max_ (MPa)	*ε*_b_ (%)
PLC-65-10.8-80	24 ^a^	153	20.7	34	22.3	560 ± 35	12.4 ± 0.6	134 ± 76
PLC-56-5.4-145 ^b^	−10	60–150	6.3	10	6.8	36 ± 3	30 ± 5	905 ± 51
PLC-58-4.8-156 ^b^	−7	70–140	4.3	7	4.7	19 ± 3	6.5 ± 4.4	920 ±327
PLC-57-7.4-91 ^c^	−12	156	11.0	17	11.8	51 ± 5	2.6 ± 0.1	505 ± 37
PLC-62-7.2-180 ^b^	−19	65–145	13.9	21	14.9	68 ± 4	40 ± 6	620 ± 40
PLC-56-5.5-182 ^b^	−9	65–130	7.137	14	7.7	16 ± 2	19 ± 7	692 ± 74
a54-PLC-62-6.6-159 ^b^	−3	65–140	13.7 ± 5.1	24	14.7	60 ± 36	32 ± 12	739 ± 143
a9-PLC-56-6.4-149 ^b^	−5	65–140	13.8 ± 4.8	26	14.8	65 ± 40	27 ± 8	652 ± 78

a: from 2nd heating run, for all other samples from 1st heating run, b: PCL crystallites observed (broad peak of low intensity, c: 1:1 (*w*/*w*) mixture of PLC-65-10.8-80 and PLC-50-3.9-101 (nomenclature values calculated). χ_c,LA_: relative crystallinity, *ϕ*_c,LA_: absolute crystallinity. *E*: Young’s modulus, σ_max_: tensile strength, *ε*_b_: elongation at break.

**Table 3:** Thermal transitions and mechanical properties of PLC/PDLA blends.

Blend	LA (wt%)	*T*_g_ (°C)	*T*_m,HC_ (°C)	*χ*_c,LA_HC (%)	*T*_m,SC_ (°C)	*χ*_c,LA_SC (%)	ϕ_c,LA_ (%)	*E* (MPa)	*σ*_max_ (MPa)	*ε*_b_ (%)
B80-PLC-65-10.8-80-147k ^a,b^	72.4	17	112–152,177	38	207	11	30	106 ± 4	5.2 ± 1	338 ± 16
B80-PLC-65-10.8-80	72.4	10 ^c^	70–150	13.5	211	48	19.4	91 ± 3	6.9 ± 0.2	135 ± 9
B90-PLC-65-10.8-80	68.9	3 ^c^	120–155	21	210	50	22.2	270 ± 30	20.5 ± 3	472 ± 40
B95-PLC-65-10.8-80	67.2	0	110–155	20	210	48	17.2	79 ± 30	4.2 ± 1	435 ± 25
B80-PLC-50-3.9-101	59.8	−8 ^c^	169	36	170	-	14.5	5 ± 6	1.0 ± 0.1	244 ± 102
B90-PLC-57-7.4-91	61.6	−3	82,135	9	208	53	14.6	90 ± 7	10.6 ± 0.9	576 ± 60
B90-PLC-56-5.4-145 ^b^	60.6	−13	81,171	6	198	27	8.2	27 ± 1	27 ± 4	1020 ± 32
B95-PLC-56-5.4-145 ^b^	58.4	−16	65–145	8	200	22	8.7	26 ± 2	18 ± 10	889 ± 83
B90-PLC-58-4.8-156	62.6	−2	84,171	8	191	21	8.3	20 ± 2	9.7 ± 1.6	689 ± 88
B95-PLC-58-4.8-156	60.5	−2	84,172	2	191	40	4.9	12 ± 3	8.4 ± 4.1	845 ± 267
B95-PLC-62-7.2-180 ^b^	63.8	−10	70–145	15	199	54	14	73 ± 3	42 ± 11	564 ± 78
B90-PLC-56-5.5-182 ^b^	60.6	−14	67–130,171	17	189	16	12.7	26 ± 2	22 ± 7	657 ± 90
B95-PLC-56-5.5-182 ^b^	58.4	−15	70–110,172	20	187	44	15.1	16 ± 3	18 ± 4	607 ± 33
B95-a54-PLC-62-6.6-159 ^b^	63.8	−14 ± 5	70–150	19	199	52	16.6	68 ± 43	35 ± 12	676 ± 111
B95-a9-PLC-56-6.4-149 ^b^	58.4	−17 ± 6	70–150	16	198	49	14.6	74 ± 43	29 ± 11	601 ± 53

Nomenclature: bY-PLC: blend of the respective PLC (Y wt%) with PDLA 16k. a: blend with PDLA 147-k, b: PCL crystallites observed (broad peak of low intensity), c: from 2nd run as in the 1st run no clear *T*_g_ observed. *χ*_c,LA_ HC/SC: relative crystallinity of homocrystallites/stereocrystallites, *ϕ*_c,LA_: absolute crystallinity. *E*: Young’s modulus, *σ*_max_: tensile strength, *ε*_b_: elongation at break.

## Data Availability

The data that support the findings of this study are available from the corresponding author upon reasonable request.

## Supplementary Materials

The following are available online at https://www.mdpi.com/article/10.3390/nano11061472/s1, Figure S1: representative ^1^H NMR spectrum of a PLC in CDCl_3_, Figure S2: DSC thermograms, comparing PLC-65-10.8-80 and its blend B95- PLC-65-10.8-80, and comparing the 1st and 2nd heating runs of PLC-58-4.8-156, Figure S3: Correlation of the number average oligolactide sequence length *l*_LA_ with the relative HC and SC crystallinities χ_c,LA_ in the copolymers, and the absolute crystallinity *ϕ*_c,LA_ in the blends, Figure S4: Correlation of the LA content with the melting enthalpy and the absolute crystallinity. Figure S5: Comparison of WAXS spectra of PLC-65-10.8-80 and its blends with different PDLA content, Figure S6: Tensile curves of the blends of PLC-65-10.8-80 with different PDLA content, Figure S7: Correlation of the absolute crystallinity with the elongation at break and the Young’s modulus, Figure S8: Images of stretching and recovery of B90-PLC-74-15.7-253, Figure S9: Cell viability tested on extracts of PLC, Figure S10: Cell morphologies of L929 mouse fibroblasts on the negative control (A), positive control (B) and the PLC extract, Figure S11: Membrane integrity (LDH release) and metabolic activity (MTS assay) of L929 mouse fibroblasts cultured on extracts of PLC, Figure S12: Cell viability tested on extracts of PDLA16k, Figure S13: Cell morphologies of L929 mouse fibroblasts on the negative control (A), positive control (B) and the PDLA16k extract, Figure S14: Membrane integrity (LDH release) and metabolic activity (MTS assay) of L929 mouse fibroblasts cultured on extracts of PDLA16k. Table S1: Thermal transitions and crystallinity of selected synthesized PLC not suited for the desired blends. Experimental description of the evaluation of cytotoxicity.
