# Supplementary material for: Soft, Formstable (Co)Polyester Blend Elastomers
Source: Nanomaterials (Basel). 2021 Jun 1;11(6):1472. doi: 10.3390/nano11061472 (PMC8230036; doi:10.3390/nano11061472)
Supplement: Supplementary file 1 [file nanomaterials-11-01472-s001.zip › nanomaterials-1235986-supplementary.pdf]

Supplementary Materials to:

Soft, Formstable (Co)Polyester Blend Elastomers

By Axel T. Neffe[1], Victor Izraylit[1,2], Paul J. Hommes-Schattmann[1], and Andreas Lendlein[1,2]*

1: Institute of Active Polymers, Helmholtz-Zentrum Hereon, Teltow, Germany

2: Institute of Chemistry, University of Potsdam, Potsdam, Germany

*: Email: andreas.lendlein@hereon.de

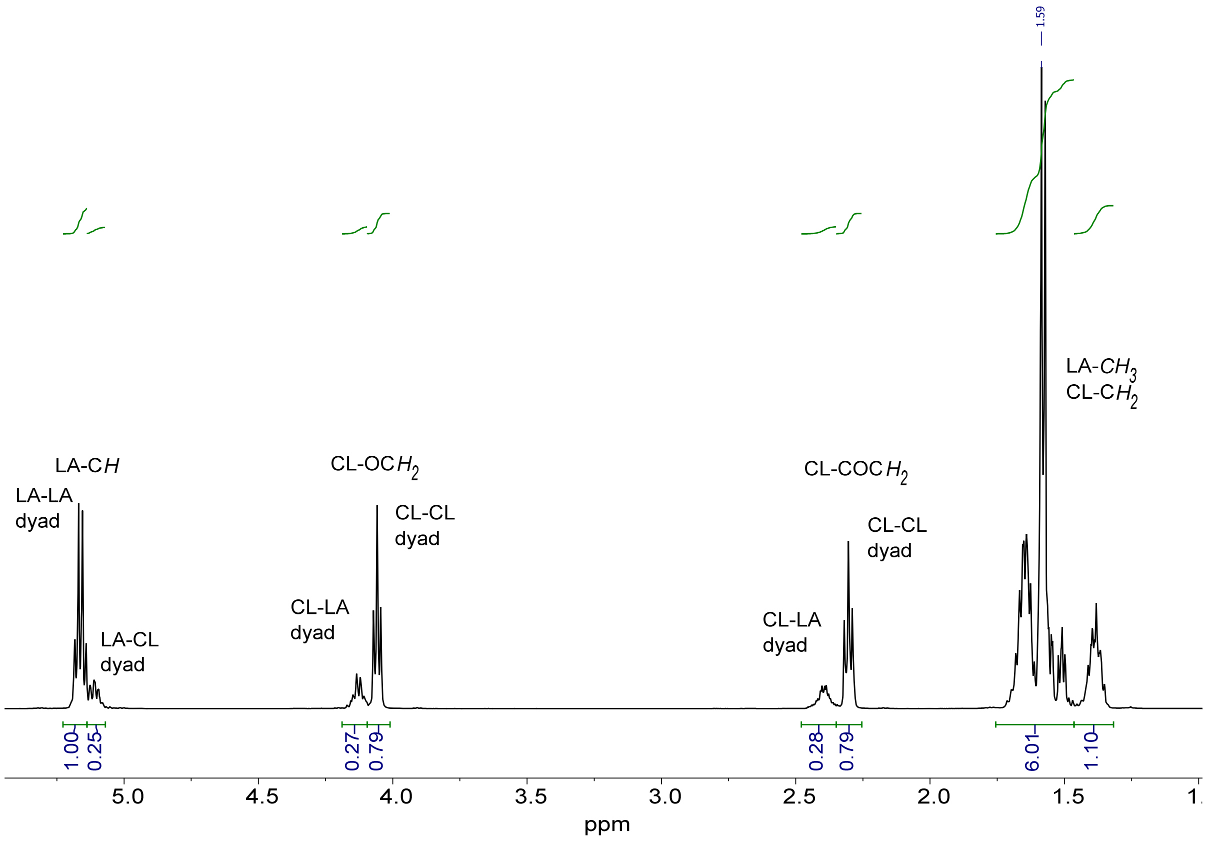

Figure S1: Representative region of a ^{1}H NMR (500 MHz) spectrum of a PLC in $CDCl_3$, with assignment of the peaks relevant for the determination of composition and sequence structure.

Table S1.Thermal transitions and crystallinity of selected synthesized PLC

Sample ID	T_m [°C]	ΔH_m [$J \cdot g^{-1}$]	$X_{c,LA}$ [%]	T_g [%]
PLC-84-24.0-49	160	44.1	56	35[a]
PLC-82-21.1-51	110, 160	39.8	52	40[a]
PLC-57-5.9-56	129	9.5	18	0[a]
PLC-57-4.9-103	n.o.	n.o.	n.o.	-2[a]
PLC-50-3.9-101	n.o.	n.o.	n.o.	-9[a]

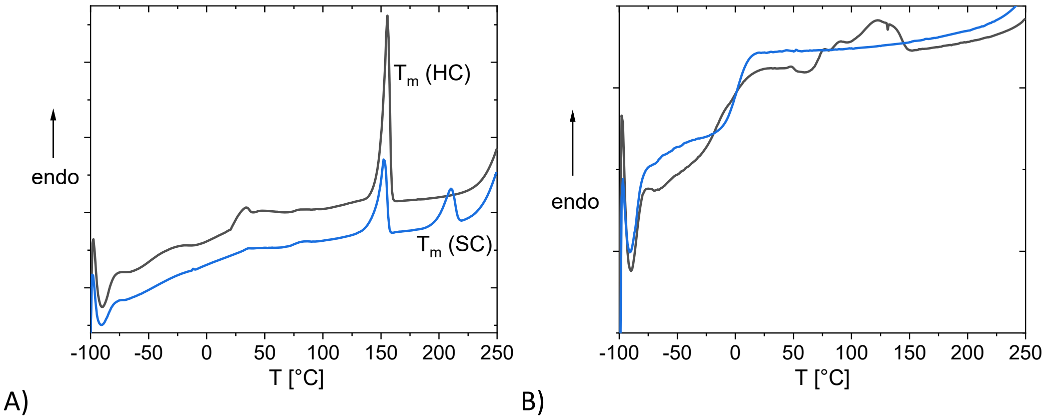

A) B)

Figure S2. A) Comparison of DSC thermograms (1st heating run) of PLC-65-10.8-80 and the blend B95-PLC-65-10.8-80. In the blend, a second melting transition indicative for stereocomplex formation is observed. The lactide crystallinity is very prominent due to the long $l_{(LA)}$ of PLC-65-10.8-80. B) Comparison of the 1st (black) and 2nd (blue) heating run for PLC-58-4.8-156. Lactide re-crystallization did not occur in the experiment, leading to a higher T_g in the second run.

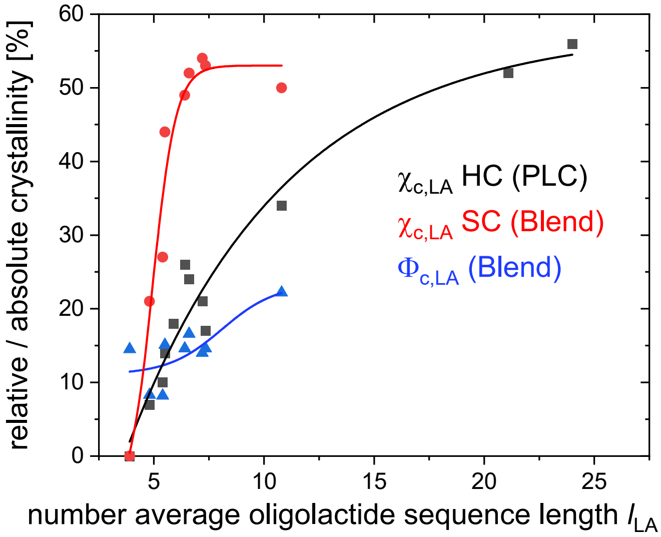

Figure S3: Correlation of the number average oligolactide sequence length l_{LA} with the relative homocrystallinity $\chi_{C,LA}$ HC in the PLC copolymers, the relative stereocrystallinity $\chi_{C,LA}$ SC in the blends and the absolute crystallinity $\Phi_{C,LA}$ (LA HC + SC) in the blends. The lines are added as a guide to the eye. In fact, the data points can be fitted well to a Boltzmann equation (R^2 = 0.76-0.98), without a known physical relation that would suggest such a behavior.

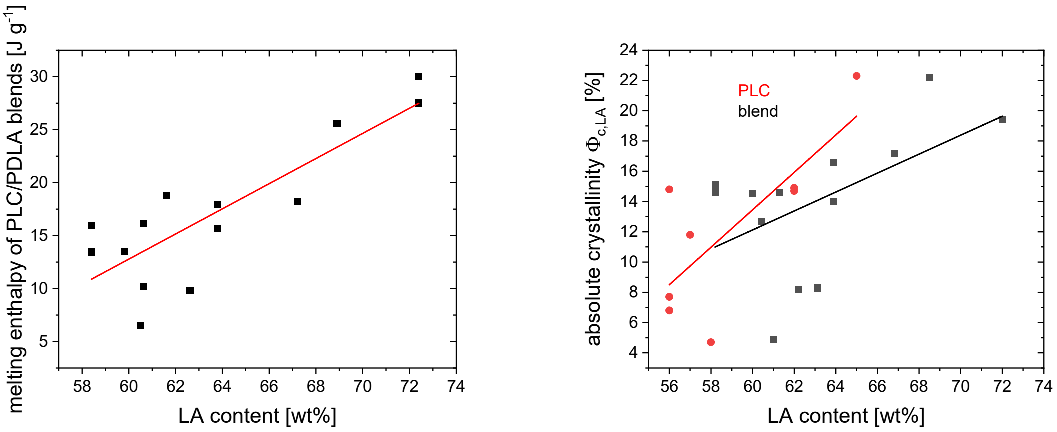

Figure S4: Correlation of the LA content with the melting enthalpy and the absolute crystallinity. The lines are added as a guide to the eye.

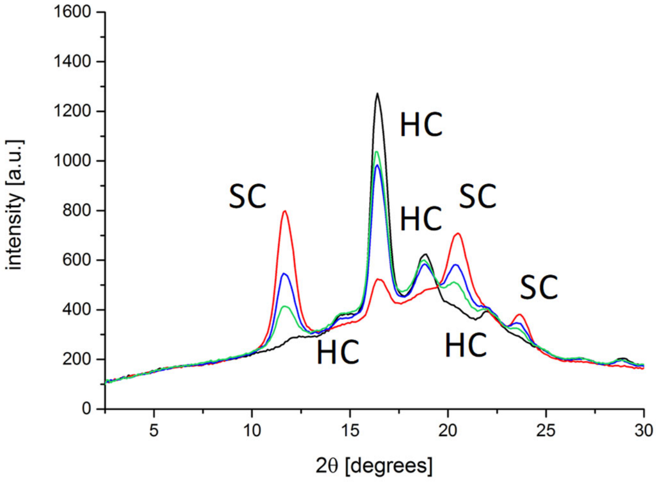

Figure S5: Comparison of WAXS spectra of PLC-65-10.8-80 (black) and its blends B95- PLC-65-10.8-80 (green), B90- PLC-65-10.8-80 (blue) and B80- PLC-65-10.8-80 (red). With increasing PDLA content, the peaks indicating stereocomplexes increased, while homocrystallite signals decreased.

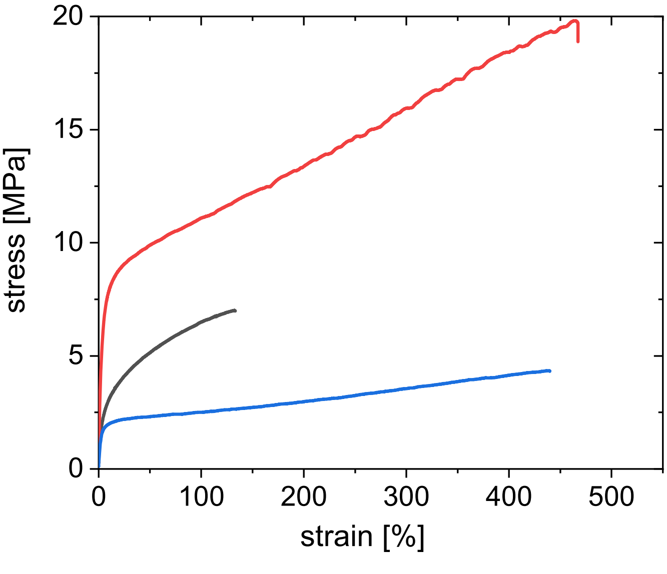

Figure S6: Tensile curves of the blends B95- PLC-65-10.8-80 (blue), B90-PLC-65-10.8-80 (red) and B80-PLC-65-10.8-80 (black)

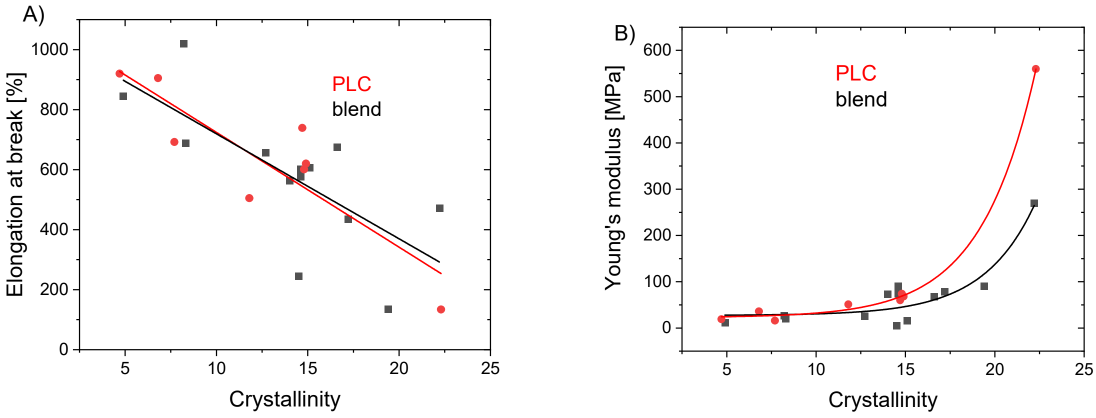

Figure S7: Correlation of the absolute crystallinity with the elongation at break (A) and the Young's modulus (B). The lines are added as guide to the eye.

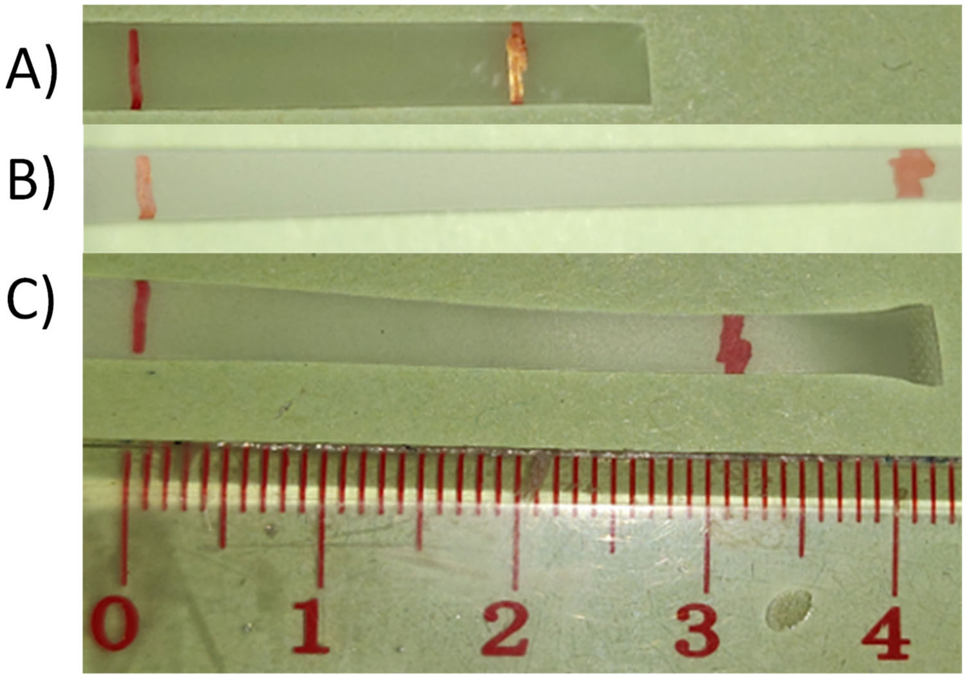

Figure S8. Stretching and recovery of B90-PLC-74-15.7-253 Overall PLA content of the blend: 76.8 wt%. A) original length, B) stretched, C) recovered after stretching.

Evaluation of Cytotoxicity

Culture of L929 cells

According to the international standard EN ISO 10993-5 cytotoxicity tests were performed with cells of a 100-day-old male C3H/An mouse (L929 cells; provided by the American Type Culture Collection, ATCC, Wesel, Germany). For cell expansion continuous subcultures of these cells were maintained under standard conditions (humidified atmosphere, 5 vol-% CO_2 in air, 37 °C; NuAire, Incubator, Germany) on polystyrene using minimal essential medium (MEM Biochrom, Berlin, Germany)

supplemented with 10 wt-% horse serum (ATCC, Wesel, Germany) and 1 wt-% penicillin/streptomycin/glutamine (Invitrogen, Darmstadt, Germany). Every second day, the culture medium was changed. The cells were subcultured when they reached a subconfluence of about 80% by rinsing them with phosphate buffered saline, and by subsequent trypsinization using trypsin-EDTA solution (trypsin 0.25 wt-% and ethylene-diaminotetraacetic acid 0.53 mM).

Cytotoxicity tests

The tests were performed using in vitro cell tests under static conditions in accordance with the supplier's instructions and in conformity with the EN DIN ISO standard 10993-5. Polymer extracts were prepared by incubating 2 g of polymer with 10 mL serum-free cell culture medium (MEM) under permanent stirring at 37 °C for three days. The resulting extract was then used as cell culture medium for L929 cells. L929 cells were seeded on a polystyrene based cell culture 96-multiwell plate (Greiner Bio-One, Solingen, Germany). After reaching a subconfluent cell layer of about 50%, the culture medium was replaced by the extract. 48 hours later the viability of the cells, integrity of the cell membrane, mitochondrial activity, and cell morphology were analyzed.

Mitochondrial function studies

As many cell processes require the activity of mitochondria, a measurement of the activity of mitochondrial dehydrogenases helps to establish the level of cell activity. The activity of mitochondrial enzymes of the cells was measured by use of a tetrazolium compound 3-(4,5-dimethylthiazol-2-yl)-5-(3-carboxymethoxyphenyl)-2-(4-sulfophenyl)-2H-tetrazolium (MTS), which when reduced produces a colored formazan product that is soluble in tissue culture medium (CellTiter® 96 Aqueous Assay, Promega, Germany). The absorbance of the colored formazan product was measured at 492 nm using a SpectraFluor Plus (TECAN, Männedorf, Switzerland) spectrophotometer.

Lactat dehydrogenase (LDH)-release measurement

The activity of the cytoplasmatic enzyme lactate dehydrogenase (LDH) was measured by use of a colorimetric assay in the cell culture supernatant (Cytotoxicity Detection Kit (LDH), Roche, Germany) to evaluate the integrity of the cell plasma membrane at 492 nm using a TECAN SpectraFluor Plus spectrophotometer (TECAN, Männedorf, Switzerland). LDH is located solely within the confines of the cell. The appearance of LDH in the cell culture supernatant therefore indicates that the integrity of the cell membranes has been compromized.

Cell morphology assessment

The morphological phenotype of the cells was evaluated according to the USP 23-NF18 (US Pharmacopeial Convention) and ISO 10993-5 using a phase contrast microscope in transmission (Zeiss, Jena, Germany) based on the cell shape and the organization of the cell layer. The number of cells on the samples was calculated as mean cell number from five different fields of view.

Endotoxin content

The eluates were analyzed for LPS contaminations using the LAL test (Lonza, Cologne, Germany), which was performed according to manufacture instructions.

Results for a PLC:

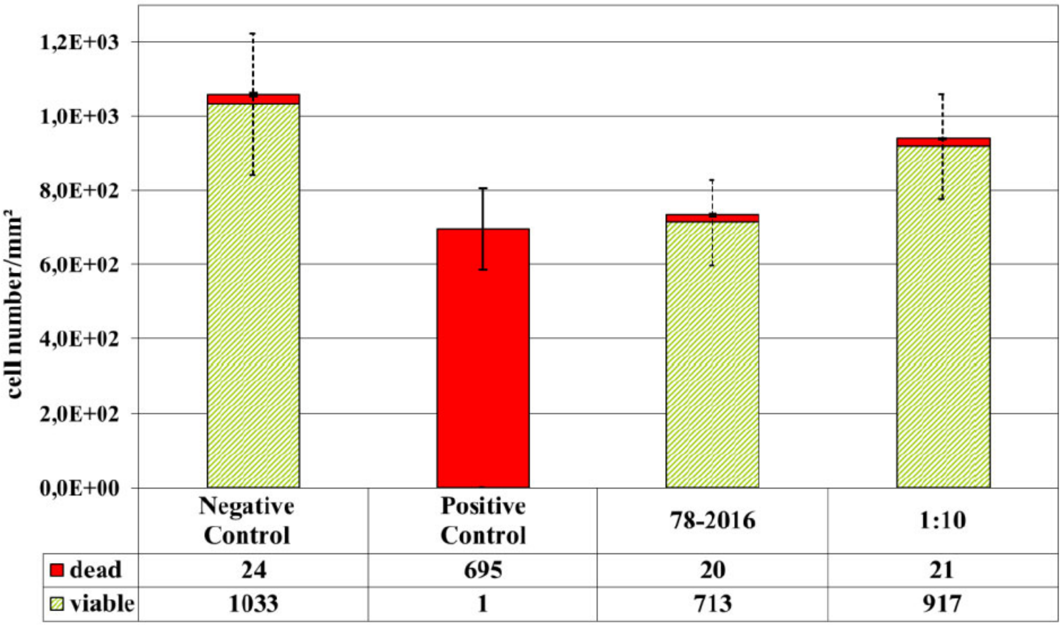

Figure S9: Cell viability tested on extracts of PLC.

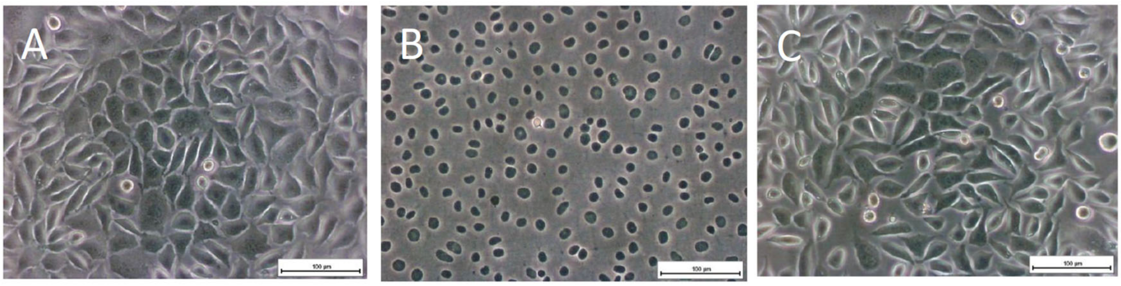

Figure S10: Cell morphologies of L929 mouse fibroblasts on the negative control (A), positive control (B) and the PLC extract.

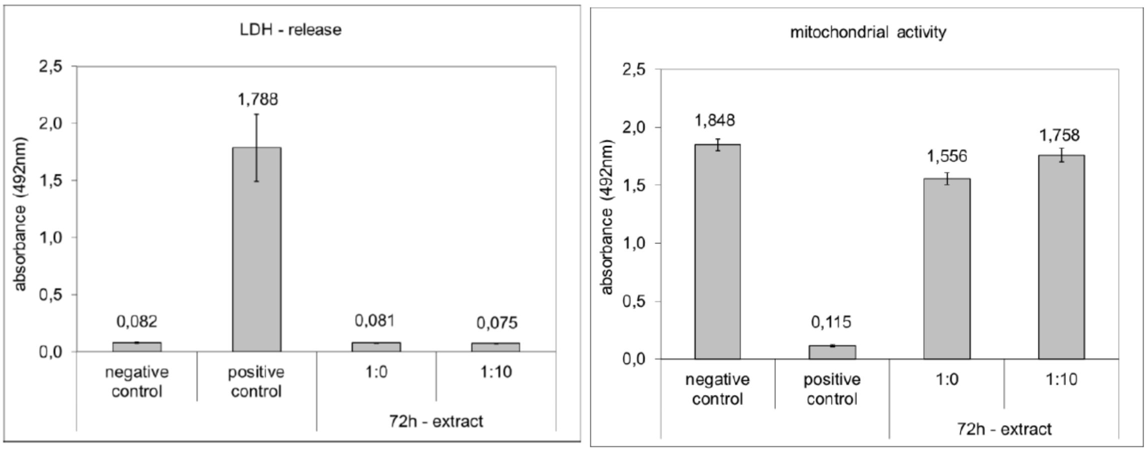

Figure S11: Membrane integrity (LDH release) and metabolic activity of L929 mouse fibroblasts cultured on extracts of PLC.

Results for PDLA-16k:

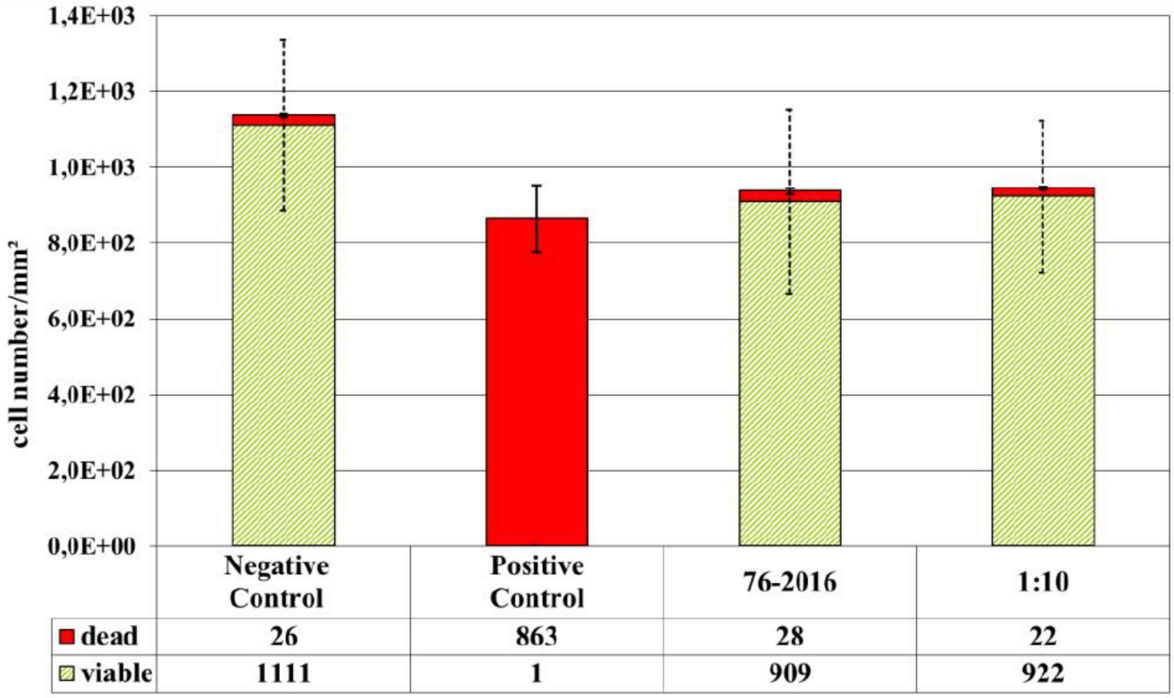

Figure S12: Cell viability tested on extracts of PDLA16k.

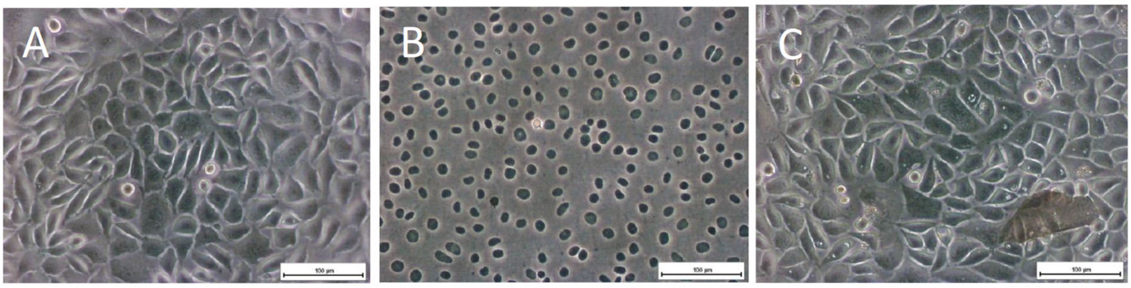

Figure S13: Cell morphologies of L929 mouse fibroblasts on the negative control (A), positive control (B) and the PDLA16k extract.

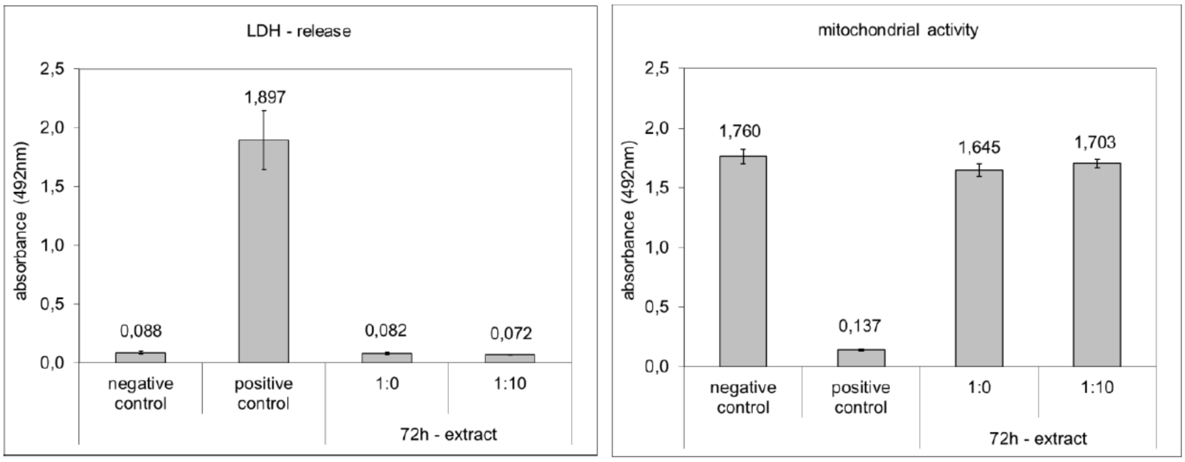

Figure S14: Membrane integrity (LDH release) and metabolic activity of L929 mouse fibroblasts cultured on extracts of PDLA16k.
